# Electronic Noses for Well-Being: Breath Analysis and Energy Expenditure

**DOI:** 10.3390/s16070947

**Published:** 2016-06-23

**Authors:** Julian W. Gardner, Timothy A. Vincent

**Affiliations:** School of Engineering, University of Warwick, Coventry, CV4 7AL, UK; T.A.Vincent@warwick.ac.uk

**Keywords:** VOC, breath analysis, energy expenditure, well-being

## Abstract

The wealth of information concealed in a single human breath has been of interest for many years, promising not only disease detection, but also the monitoring of our general well-being. Recent developments in the fields of nano-sensor arrays and MEMS have enabled once bulky artificial olfactory sensor systems, or so-called “electronic noses”, to become smaller, lower power and portable devices. At the same time, wearable health monitoring devices are now available, although reliable breath sensing equipment is somewhat missing from the market of physical, rather than chemical sensor gadgets. In this article, we report on the unprecedented rise in healthcare problems caused by an increasingly overweight population. We first review recently-developed electronic noses for the detection of diseases by the analysis of basic volatile organic compounds (VOCs). Then, we discuss the primary cause of obesity from over eating and the high calorific content of food. We present the need to measure our individual energy expenditure from our exhaled breath. Finally, we consider the future for handheld or wearable devices to measure energy expenditure; and the potential of these devices to revolutionize healthcare, both at home and in hospitals.

## 1. Introduction

There are few ways to measure our well-being without subjective surveying. Our own health, of course, contributes significantly to us feeling well and can provide a tangible measure of our quality of life. There is an urgent need for a method to monitor the health of a population where our sedentary lifestyles threaten our well-being. Diagnoses or prevention of ill-health is a multi-million dollar market, where obesity is ranked today as the third highest global economic burden. Electronic noses are poised to capture a hold in this sector of the healthcare industry, as analysis of human odours offers an unrivalled ability to monitor the health of a person. For example, the molecular composition of our breath can provide a measure of our energy expenditure (EE), as well as the alcohol present in our blood; it can aid the diagnosis of diabetes (ketones) or cancer (biomarkers) and can help identify respiratory diseases, such as asthma (nitric oxide). In this article, we will explore the wealth of information available through the analysis of exhaled breath measurements captured with simple chemical sensors, rather than expensive analytical equipment, such as gas chromatograph mass spectrometers.

In the UK alone, it is estimated there are 5.5 million people are categorised as obese [1], and one million are classified as morbidly obese (double that of two decades ago) [2]. Over the same period, our working and leisure activities have changed, leading to the majority of us spending 6–7 h per day sedentary, i.e., idle. It is estimated that treatments related to an overweight population cost the British National Health Service £15.4 billion ($21.9 billion) a year, an expense that increased from £5.1 billion in July 2006 [3]. Obesity has a staggering financial impact on the U.K. of $73 billion [4]. This enormous outlay could potentially be avoided, if people were educated and guided towards a healthier lifestyle. Over eating, a principal cause of obesity [5], is difficult for a person to self-diagnose [3], but treatment is neither costly nor complex. The calorific intake required for an individual can be calculated through equations, such as the well-known Harris-Benedict [6] or Mifflin St Jeor [7]. Such formulae rely heavily on the weight and height of a patient, thereby neglecting numerous characteristics of an individual, and usually, equations are unable to predict requirements for ill patients. Breath analysis offers a proven solution to determine the energy requirements for a subject, based on the energy they are consuming. The energy expenditure (EE) of a patient, the amount of energy consumed at a given instant in time, can be used to advise daily calorific intake.

EE is often monitored over a 24 h period, thus accounting for the variations in metabolism throughout the course of our daily activities. The Weir Equation (1), states that the EE of a subject can be related to the volume of oxygen consumed ${\dot{V}}_{O_{2}}$ and the volume of carbon dioxide produced ${\dot{V}}_{CO_{2}}$ [8].

(1) $$EE\;\left[\frac{kcal}{day}\right]=3.9\left({\dot{V}}_{O_{2}}\right)-1.1\;\left({\dot{V}}_{CO_{2}}\right)$$

*Respiratory rooms* are the gold standard of measuring EE through indirect calorimetry, where a patient resides in a chamber for the measurement period [9]. The advancements in sensor design in the decades following the conception of whole body calorimeters has led to the development of portable measurement systems [10]. We discuss the most recent metabolism monitors and the future possibilities of smart, affordable and mass-market devices.

*Breath analysers* are already recognised as diagnostic equipment for a number of respiratory disorders; but their use can extend to indirect diagnoses through biomarker detection. Below, we will discuss how breath tests can be used to detect gastric infections, monitoring asthma therapy, monitoring diabetes, alcohol consumption and neonatal jaundice. The measurement or detection of VOCs forms an integral part of identifying a specific disease. Over 300 different VOCs have been found in exhaled breath. Indeed, examinations of breath samples have been reported for the purpose of detecting lung, breast, colorectal and ovarian cancers. Arrays of sensors are usually required to detect the low concentration of a specific VOC (often in the ppb range), and pattern recognition algorithms are used to classify the response to a particular condition.

## 2. Non-Invasive Measurements

The concept of sampling breath for health monitoring was initially conceived in the 20th century. In 1952, Henderson reported on the increased acetone content of breath samples from young diabetics, promoting an interest in the content of breath [11]. Exhaled breath can provide a non-invasive alternative means of investigating the substances found in the blood. Proportional concentrations of compounds present in the blood are also present in air expired from the alveoli. For a 70 kg male, the initial two thirds of an exhalation comprises gases from dead-space volumes, and it is only the final 250 mL that contain the desired *alveoli gas* [12].

Electronic noses (or E-noses) in the past have been mainly restricted to laboratory environments and unfortunately not in the real world as desired for ubiquitous breath sampling. Breath gas is not the easiest substance to store, where containers must be capable of holding an exhalation, but also prevent the contamination/ageing of a sample. No standard exists for collecting breath [13]. Sampling systems have been designed in the past to separate the desired end-exhaled portion of a breath. The exhaled gas desired is found from an exhalation to be distinguishable from the initial gas when it reaches 6% absolute humidity or body temperature (~37 °C) [14]. Mechanical collection systems often work by discarding a volume of air, before capturing the end-exhaled portion (perhaps 150 mL) [15]. Hand-held portable E-noses are becoming possible, with advances in sensor design allowing for compact, power-efficient and reliable sensors.

Breath is a non-invasive means of diagnosis and preferred over blood and urine specimens, which can be tedious and cumbersome to analyse, particularly for VOC compounds [16]. Samples are easily obtained and can be provided immediately. However, it is noted that the samples must usually be analysed with E-noses or the like, whereas urine measurements can be used with simple chemical tests.

In terms of EE measurements, *whole body calorimeters*, measuring the gaseous contents of a room with O_2_ and CO_2_ sensors, is recognised as the “gold standard” [17]. The EE of a subject is monitored, perhaps on a minute by minute basis, through indirect calorimetry. In some cases, the nitrogen excreted through urine is also recorded, although it was found that discarding these measurements only added a small error of 1%–2% for inpatients and outpatients [18]. Indirect calorimetry can also be performed using metabolic karts, which provide a bulky, but portable measurement system, which are usually confined for use in hospitals. A great advantage of breath measurements is that they are completely non-invasive; however, with the current generation of sampling techniques, it is also one of their weakest attributes. To obtain a complete overview of the EE of a subject, they should be in their free living environment, unhindered by the measurement procedure. This is an impossible notion inside a whole body calorimeter, where the subject must be isolated, even from medical staff. Portable analysers also hinder the movement of the subject, where they are restricted to having to wear a mask.

Over longer periods of time (up to three weeks), the *doubly-labelled water* (DLW) method is the “gold standard” for assessing total EE [19]. DLW measurements only provide a single measure of EE per sample taken, so they cannot provide an output of how EE varies throughout the course of a single day, nor a comparison between diurnal cycles of, for example, weekends or weekdays. The technique is commonly used for validating questionnaire studies, such as food-frequency or physical activity. A two-week food-frequency study is reported by Kroke, noting that out of 30 subjects in general, energy intake recorded by a checklist questionnaire was under-reported [20]. A review by Hill notes a generalised underreporting of food intake in many subject groups [21]. There is a need for a standardised technique, accessible to the general population, for EE monitoring. The calorific content of snacks and alcoholic beverages is usually underestimated [3], the over consumption of which has been linked to obesity [22].

Heart-rate monitoring has been reported as a non-invasive technique to calculate the EE of a free-living human subject [23]. A notable technique, coined *Flex HR* by Spurr [24], offered a standard means of converting minute by minute heart rate measurements into EE information. The study performed by Spurr demonstrated a variance of only +2.7% compared to indirect calorimetry. The technique requires careful calibration and is somewhat fundamentally flawed, as not all heart rate variation is due to metabolic activity [25]. The Flex HR method was verified by others [26,27] as producing results within ±2% of whole body indirect calorimetry. Where heart rate tracking offers comparable results for “adult” subjects, comparisons of the Flex HR to respiratory chambers with groups such as children and lactating women have proven that the technique cannot offer reliable resultants across a varied population. Errors in the order of 16.0% ± 13.8% and 2.0% (range −22.2%–52.1%) were reported [28,29]. Similarly, studies by Tanhoffer and Rothenberg comparing Flex to DLW with elderly subjects and patients with spinal injuries have also demonstrated errors of −9.7% and +13% [29,30].

## 3. Breath Contents

There is growing concern about the levels of harmful VOCs we inhale in our workplaces, homes and cities. VOC detectors are available to assess the air quality in outdoor environments and to warn of any health risks. In the same way, the VOCs and gas contents of our exhaled breath offer a promising means to diagnose diseases [31,32] and can also contribute to evaluating our lifestyles. In this section, we discuss the gases and VOCs found in breath and their use in healthcare applications.

### 3.1. Exhaled Gas

The primary function of the alveolar capillary interface, inside a human lung, is to exchange oxygen and carbon dioxide [33]. However, in terms of medical potential, the characteristics of each exhalation, as well as its content, can highlight the symptoms of diseases and provide a means of real-time monitoring. Table 1 lists examples of gases reported in breath samples and their significance to healthcare.

The carbon dioxide content of breath is often monitored and displayed graphically (capnography) [59]. Repetto reported the improvement in endotracheal tube placement time, by the use of capnography to detect when the tube was in the trachea. The correct placement of the tube could be verified by observation of the capnograph [59]. In the case of a clinical trial with 16 infants, the placement time decreased from 35 s to 9 s. It is suggested that the end tidal CO_2_ (ETCO_2_, maximum level of CO_2_ at the end of an exhalation) level is used to guide ventilator management [36]. Continuous *capnometry* (the numerical display of CO_2_ concentration) is suggested during the transport of mechanically-ventilated patients [36,60]. Capnography is suggested to identify the abnormalities of exhaled air flow [36,61,62].

Carbon monoxide (CO) is often associated with smoking and the numerous possible diseases patients who smoke can contract. The detection and quantification of the CO content from a breath sample can increase the awareness of the potential harmful consequences of a smoking habit [37]. Deveci demonstrated that healthy smokers exhaled 17.1 ppm CO on average, over a sample size of 243 subjects. Carpagnano tested 21 smokers and similarly found that the average CO content of exhaled breath was 16.7 ppm [63]. On average, Carpagnano reported that 14 non-smokers exhaled 2.1 ppm. Deveci reported a higher level of 3.6 ppm for breath samples from non-smokers (although noting that the pollution in the city environment from which subjects were sourced could account for the higher than expected level). Deveci sampled 24 passive smokers, subjects who themselves were non-smokers or had not smoked for over 10 years, but who were exposed to an environment of tobacco smoke. The passive smokers on average exhaled 5.2 ppm CO, noted as not significantly higher than non-smokers. In general, the cut-off for distinguishing between smokers and non-smokers is taken as ~6 ppm [39].

CO levels in the range of 6–10 ppm in non-smokers could indicate the presence of a respiratory disease or perhaps indicate that the subject was not disclosing his or her smoking habit [38]. In addition to their use as quantifying smoking habits, CO analysers could also indicate numerous inflammatory lung diseases, such as bronchiectasis, asthma and primary ciliary. Middleton reports on the case of a patient who gave samples of >6 ppm CO, however claiming not to have smoked. Middleton suggests it would be prudent to confirm smoking status with a urinary measurement, else the increased CO exhalation level could be due to COPD.

The CO content in breath samples from asthmatic patients is notable higher than non-smoking subjects (~5.6 ppm untreated group), but is close to the cut-off level between smokers and non-smokers. Zayasu reported on 110 subjects; 30 subjects in each group of the non-smoker control, untreated asthmatics and treated asthmatics. For a comparison, a further 20 smokers were tested. Here, the level of CO in the control group was on average 1.5 ppm, lower than the asthmatic group (treated) 1.7 ppm. The exhaled breath from smokers on average contained 21.6 ppm CO [41]. As the level of CO in untreated asthmatics is almost within the region of a subject who smokes, the test cannot conclusively categorise subjects.

Lal reported on the increased CO level detected in subjects with sickle cell anaemia (SCA) [42]. Data collected from the breath samples taken from 16 children with SCA demonstrated a median level of 4.35 ppm CO compared to 0.80 ppm for a control group. Patients who were receiving hydroxyurea produced on average 32% lower end tidal CO readings. It was suggested that the increased level of CO was caused by haemolysis, where CO production is increased from heme catabolism. The CO by-product offers the possibility of detecting non-respiratory-related diseases. Zegdi reports on the increased CO production in patients with severe sepsis [40]. The increase in CO content is not significant where on average, levels of 0.54 ppm were measured in control subjects compared to 1.13 ppm for subjects (60 measurements) with severe sepsis (inhaled 0.25 ppm CO).

Nitric oxide (NO) is regarded as one of the most interesting gases in an exhalation in terms of diagnostic potential [43]. Berkman presented the need for a standardised NO test that could replace the time consuming and costly bronchial provocation tests [44]. No single provocation test is considered the “gold standard”, and all such tests present the risk of bronchospasm, which could be avoided with a breath gas test. In the study, 85 patients provided breath samples, 40 asthmatic and 45 non-asthmatic. The median exhaled NO concentration was close to four-times the normal value versus asthmatic (5.3 ppb and 19.2 ppb, respectively). Berkman concluded that the NO test produced comparable results to any prior conventional bronchial provocation tests. The cut-off level was selected at 7 ppb to distinguish asthmatics with a specificity of 95.6%. Pedrosa reports on a similar experiment with 114 participants [46]. Thirty five subjects were diagnosed as asthmatics, with median exhalation concentrations of 30 ppb NO. It is noted that nearly half the patients had concomitant rhinitis symptoms. Patients in this group provided median breath NO levels of 39 ppb.

A higher NO exhalation level than healthy subjects cannot be used for conclusive diagnosis of asthma. Table 1 lists three examples of other diseases that also affect the NO concentration on breath. Particularly, rhinitis could cause a false positive asthmatic diagnose, where the mean value of 16.3 ppb NO, reported by Martin [47], is above the asthmatic cut-off levels suggested by Berkman (7 ppb) and Dupont (13 ppb) [64]. Martin tested 18 control subjects and 32 patients with seasonal rhinitis (grass pollen-sensitive). Martin also found a breathing hold produced higher NO concentrations in patients (62.0 ppb after holding for 60 s), and a nasal breath demonstrated 35.4 ppb on average.

Murphy reported on increased NO exhalation levels due to an induced influenza infection [48]. The study tested the relationship between exhaled NO in healthy volunteers given a viral infection. Breath samples were taken on days 0–4, 8 and 21 by collecting samples in Mylar balloons. Exhaled NO levels decreased slightly over the first four days, after inoculation of the virus, and peaked on day 8 (mean 12.9 ppb). By the follow up breath test on day 21, the exhaled NO level had returned to baseline. The volunteers were kept in isolation for eight days. Symptoms scores peaked on day 3; however, exhaled NO levels remained close to baseline. Murphy concludes that the variation of exhaled NO levels suggests that NO does not contribute directly to illness manifestations. However, the cause of the elevated level of NO on Day 8 is not clear, where patients had no respiratory tract complaints, and when kept in isolation, the chance of contracting another illness to elevate NO is unlikely.

*Cystic fibrosis* (CF) is associated with respiratory infection and inflammation. Balfour-Lynn studied the exhaled NO concentration in breath samples from a group of 63 children diagnosed with CF [50]. Considering that respiratory diseases, such as asthma, increase the level of NO found in the breath, Balfour-Lynn notes surprise upon finding that CF has the opposite effect. The CF patients were split into two groups, those prescribed steroids and those who had not taken steroids for six weeks. The 50 subjects on steroid medication exhaled on average 3.6 ppb NO, compared to the average of 4.7 ppb for the 13 subjects not on steroid medication and 4.8 ppb for the control group (57 children). NO cannot be used for monitoring lung inflammation in CF patients. The decrease in the concentration of NO exhaled may contribute to the recurrent bacterial respiratory infections often suffered by such patients, considering the antibacterial nature of NO [50]. The NO breath concentration for the CF group without steroid medication was only 0.1 ppb lower than the control group. A comparison of the average values found by the study could prove erroneous, where the breath NO concentrations were measured using an instrument with a 1-ppb resolution limit. The findings reported by Barflour-Lynn are supported by Graseman, similarly recording a decreased NO level on the exhaled breath from 75 CF patients [49]. The mean exhaled NO level was 2.3 ppb, where the patients had been instructed not to take steroids for at least two weeks prior to the study.

Hydrogen and methane breath tests are approved by the U.S. Food and Drug Administration (FDA) [65]. The tests are used to detect gastrointestinal transit time, bacterial overgrowth and intestinal status. The extensive use of these tests in clinical practice demonstrates their potential for diagnosis, but the results need careful interpretation to avoid erroneous conclusions [52,53,54]. When fasting, healthy humans at rest do not exhale hydrogen. A basal measurement is taken prior to any experiment (usually <5 ppm). A test substance (e.g., fructose, lactose, glucose) is administered to the subject. If the subject produces excess hydrogen (>20 ppm increase above basal value), this is considered a positive test [52]. Hydrogen is produced in high quantities if there is a malabsorption of the test substance. There is undoubtedly diagnostic potential with the hydrogen test; however, the results of the investigations must be carefully scrutinised to avoid misinterpretation or, worse, abuse of the test [53]. Table 2 lists some possible interpretations for three hydrogen production tests.

The production of relatively high levels of methane in the breath can indicate *mal-absorption* of a substance. It is feasible a patient could provide a false negative hydrogen test result, but still suffer from mal-absorption. It has been reported that methanogens occur in about one third of adult humans [56]. It is rare for a human to be a co-producer of both methane and hydrogen; thus, a hydrogen breath test cannot conclusively diagnose mal-absorption. It has been reported that a healthy adult will produce a low concentration of methane (1 ppm above atmospheric [57]). The mean concentration of methane producers was 16.6 ppm above atmospheric as found by Levitt. In a similar way to the hydrogen tests above, methane producers can react to substances, such as lactose and fructose [56]. A high concentration of methane in exhaled breath can help diagnose diverticulitis, constipation or irritable bowel syndrome [57,58].

### 3.2. Exhaled VOCs

VOC detection and the use of electronic noses are of great interest in the field of breath analysis research for disease diagnosis. Where the detection of an abnormal quantity of one compound or gas in the breath of a patient can help a diagnostician reduce possible causes, the identification of several abnormal levels of compounds or gases can further a diagnoses, as there are almost no unique VOCs for a disease [66]. The current generation of research breath sensing systems are often sensitive to a range of compounds, with the target to design an ultimate diagnostic tool, where a specific disease can be recognised from a “fingerprint” of exhaled substances. Table 3 lists recent examples of the biomarker VOCs with the greatest potential to differentiate breath samples from patients diagnosed with a disease and control subjects.

COPD is a progressive and debilitating disease; thus, early detection improves the prognosis of suffers. VOCs have been widely linked to respiratory diseases, but a specific detection method based on the analysis of VOCs for COPD has not yet been developed [70]. Gas chromatography is considered the gold standard for air analysis. GC-mass spectrometry is widely used for the analysis of exhaled breath. In the COPD studies listed in Table 3, all use a form of GC to identify VOCs [67,68,69]. Cazzola compares a GC-MS to an E-nose consisting of an array of six quartz microbalances, coated with 5,10,15,20-tetra-kis-(4-alkyloxphynyl) porphyrins [70]. The coating of each sensor is complexed with a different metal, to produce a correlated frequency shift depending on the VOCs present. A cross-validated PLS-DA model classified the subjects. The model correctly classified 26 of 27 COPD patients and five of seven control subjects.

Lung cancer is perhaps the most studied disease from the VOCs in breath. E-noses have been developed with the aim of a quick diagnosis; however, there has yet to be a standard detection method. The range of VOCs presented in Table 3 reported to have been detected in the breath from lung cancer patients demonstrates that no one set of compounds has been identified as the ‘fingerprint’ for the disease [92]. In a similar manner to COPD detection, E-noses for lung cancer detection are often based on either quartz microbalances or surface acoustic wave resonators, with the advantage that such systems can be used for *chemical fingerprinting*. GC-MS is frequently used to verify research sensor arrays. Di Natale first reported on an array of eight quartz crystal microbalances coated with different metalloporphyrins [93]. The sensor system outputs were classified using a partial least squares method and correctly identified 100% of the lung cancer patients (35 tested). A total accuracy of 90.3% was achieved, considering that the system was less able to separate the post-surgery and the healthy control groups. Gasparri further reports on the development of a matrix of eight microbalances, with a volunteer group size of 146 subjects, 70 of which had been confirmed lung cancer patients by tomography [94]. “Breathprints” of lung cancer patients were differentiated from those of the healthy control group with a sensitivity of 81% and a specificity of 91%.

Surface acoustic wave (SAW) sensors can also be effective in the recognition of lung cancer patients. Chen reported on a pair of SAW sensors (one reference, one polymer coated) operating at 52 MHz [75]. The E-nose was calibrated against known VOC concentrations, chosen based on the data of exhaled VOCs from 20 lung cancer patients (collected using an MS). The calibrated system was tested against 10 subjects, 5 patients and 5 controls. The system correctly identified four of five lung cancer patients and four of five healthy controls. The two incorrectly identified were classified as “suspected”. Rudnicka compared the identification of lung cancer using chromatography-MS against trained dogs [71]. One hundred and eight lung cancer patients were tested and compared against 121 healthy volunteers and 24 people with other lung diseases. The model to predict lung cancer using MS data was found to have 74% sensitivity and 73% specificity, compared to 86% and 28%, respectively, for the trained dogs.

In the United States, breast cancer is the second most common cancer among women [79]. Xu reported on a sensor consisting of an array of nanowire sensors on a chip for the detection of breast cancer. Four biomarkers were chosen for this application, heptanal, 1-phenyl-ethanone, isopropyl myristate and 2-propanol. The system was tested on synthetic mixtures of the biomarker VOCs, and the device was found to respond to low ppm levels, close to the normal concentration of the biomarkers in exhaled breath. No pre-concentration procedure was required [79].

Pennazza reported on the *breathprinting* of liver disease using an array of seven quartz microbalances coated with anthocyanins [95]. The 104 subjects consisted of 39 patients with non-cirrhotic chronic liver disease and 65 with liver cirrhosis. The sensor system correctly classified 81.3% of cirrhosis patients. The elevation of ammonia in the breath could be used as an indicator for renal and liver disease [80]. A semiconductor-based sensor was trialled by Kao, showing promising results in laboratory conditions. It was suggested that the device could provide a non-invasive solution for the diagnoses of liver disease by using ammonia as a biomarker.

Colorectal cancer has been detected in breath using an array of gold nanoparticles [96]. MS have been used for breath, although this technique is noted as costly and time consuming [97]. E-noses are suggested as an alternative solution, offering real-time and high-throughput analysis. Faecal samples, flatulence and urine E-noses have been reported [97,98,99]. A report by Meij demonstrated a sensitivity of 85% for distinguishing colorectal cancer patients with control subjects [97]. The study consisted of faecal samples, examined with a commercial e-nose (Cyranose 320) with 32 polymer sensors. Amal reported a nano-array of six sensors based on two types of nanomaterial for use as a screening tool for colorectal cancer [87]. The array system was found to have a sensitivity of 85% and a selectivity of 94%. Acetone and ethyl acetate were found in higher concentrations in cancer patients compared to the control group. Ethanol and 4-methyl octane were higher in the control group.

An electronic nose formed of an array of electrochemical cells, an infra-red optical cell and a photo-ionisation cell has been reported with the capability to distinguish colorectal cancer patients using the headspace from a urine sample [99]. The data from an array of sensors (oxygen, ammonia, ethylene oxide, ozone, nitic oxide, nitrogen dioxide, sulphur dioxide, hydrogen sulphide, hydrogen, carbon dioxide and methane with a photo-ionisation sensor) were logged for use with a classification algorithm. It was noted that the sensor system has a high cross-sensitivity, and it was likely that other VOCs than the compounds listed would produce a response. The system achieved a sensitivity of 78% and a selectivity of 79% from a cohort of 92 urine samples. Westerbrink concludes that the system demonstrates the potential for electronic nose technologies to be used as diagnostic tools for colorectal cancer [99].

A nano-array system consisting of six sensors was reported by Amal as being able to differentiate ovarian cancer patients with healthy controls with a sensitivity of 79% (100% specificity and 89% accuracy) [91]. A report by Kahn demonstrated that an array of ten flexible molecularly-modified nanoparticles was able to distinguish between healthy controls and patients with ovarian cancer [90]. The study tested the array of sensors from a controlled gas source to verify that the devices were sensitive to the types of VOCs found in previous work using MS. Breath samples were taken from a total of 43 subjects were tested (26 control, 17 patient). The system was able to classify the volunteers with 83.4% sensitivity and 80.8% specificity (81.4% accuracy). Kahn summarises that the work raises the hope of achieving an extremely simple, inexpensive, portable and non-invasive diagnostic procedure for cancer and other diseases.

## 4. Energy Expenditure

**Figure 1:**
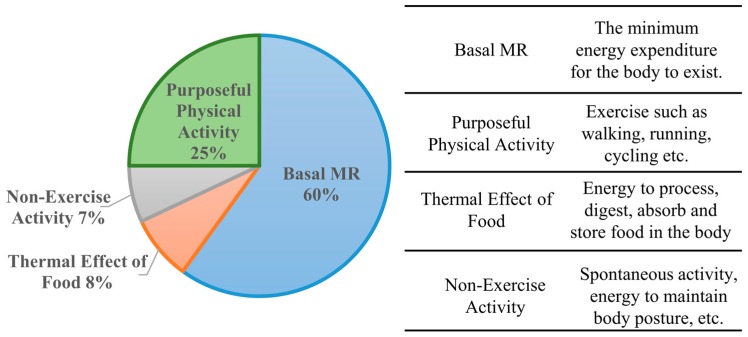
Components of EE [100].

The amount of energy burned during our daily lives varies, dependent on our activity levels and basal metabolic rate. Our EE can be divided into four components: purposeful physical activity, basal metabolic rate (BMR), non-exercise activity and thermal effect of food (TEF) [100], as shown in Figure 1. A study by Ravussin of EE over a 24 h period on 118 subjects demonstrated that the average EE was 2275 kcal/day and that the average energy intake was 2330 kcal/day [101]. On average, the daytime EE was 1.78 kcal/min, and sleeping EE was 1.12 kcal/min. The thermal effect of food was calculated as 165 kcal/day (7% of total EE), and the cost of physical activity was calculated as 348 kcal/day (15% of total EE). The lower physical activity component is likely due to the subjects being isolated inside the respiratory chambers. While the subject is enclosed inside the chambers, it is unfortunately not possible to replicate a free-living environment.

Knowledge of EE would be useful to the general population, for the management of calorific intake and lifestyle guidance [102]. Studies have already demonstrated the benefits of monitoring the EE of athletes, patients in intensive care units and obese patients. BMR is defined as the minimum amount of energy required to sustain conscious life. It can only be measured under strict laboratory conditions, where a 12 h fast is required beforehand and no strenuous activity performed for at least 1 h prior to the measurement [103].

**Figure 2:**
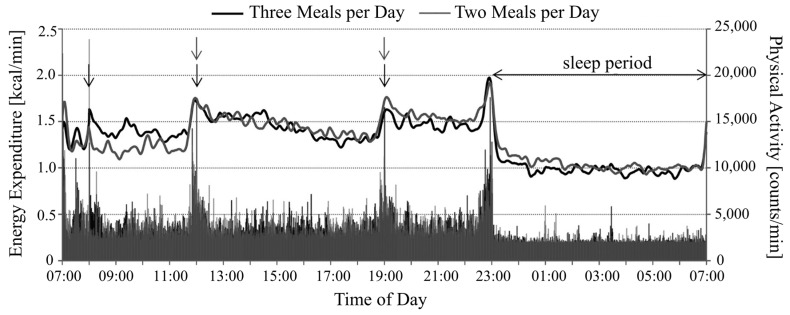
EE and physical activity measured under conditions of two and three meals per day. Arrows indicate meal times. Adapted from [107].

The TEF component of EE varies by person and is one of the more difficult methodological barriers to the study of energy balance in men [104]. Diet variation can influence TEF by as much as 15% (for a healthy subject, ~10% is expected) [105]. Oxygen consumption, which correlates between body fat, body surface area and body weight, has been shown to vary in accordance with TEF; O_2_ consumption has been reported to rise after a meal and is lowest in the morning and at night [106]. Figure 2 shows the changes in EE measured for subjects inside a chamber, one group given two meals during the experimental period and the second group given three meals [107]. TEF measurements inside respiratory chambers were noted as “not ideal” by Tataranni, due to poor reproducibility [108]. The variance in EE is close to the noise margin for respiratory chambers. TEF can have an effect on EE for up to 10-h after the last meal [109]. The energy content of the food most determines the TEF, followed by the protein factor. Relatively high protein content food and high alcohol consumption contribute to a significantly higher thermal effect, whereas food with high fat content reduces the effect.

Physical activity by definition causes an increase in calories burned, with the relationship between the energy expended and the intensity of the activity well understood. The effect of daily EE is less well reported, however. It has been proven that exercise can affect the metabolic rate for several hours, beyond the duration of the activity [110]. Knab reported that subjects displayed an increased metabolic rate for 14 h after a 45 min bout of cycling, when measured in a metabolic chamber [111]. The data from 10 subjects demonstrated during the exercise period that EE increased by on average 514 kcal, and over the following 14 h, a total of 190 kcal was expended above previous measurements. After approximately 14 h, the EE measured returned to the baseline level, set by previous days inside the chambers.

**Figure 3:**
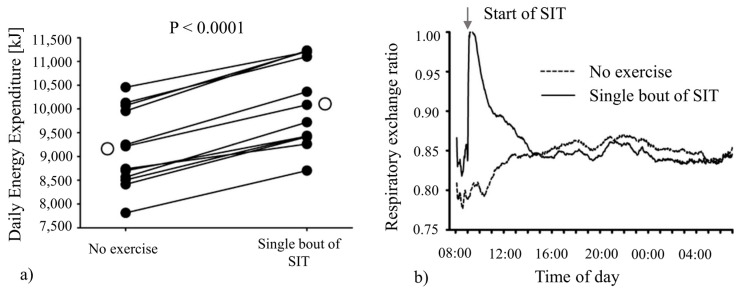
(**a**) Total daily EE increased after a single bout of SIT. Closed and open circles indicate individual responses and the group mean, respectively; (**b**) mean EE found from the group during a sedentary day compared to a day with the SIT period. Adapted from [112].

Similarly, Sevits studied the effect of a single bout of sprint interval training (SIT) on the daily EE of 12 adult males inside respiratory chambers [112]. The SIT consisted of five sprints on a cycle ergometer, with recovery time in between (total period of ~30 min). An increased level of EE was recorded for 4 h after SIT, and an increased mean daily EE of 225 kcal was calculated, shown in Figure 3a. Sevits further explored the influence of SIT on 24 h metabolism by use of respiratory data to quantify changes in substrate oxidation. It was found minute by minute that respiratory exchange ratio values, shown in Figure 3b, were increased only for a 4 h period.

The examples of studies performed in whole-room calorimeters shown above demonstrate that the rate of EE varies throughout even a sedentary day, influenced by meals (timing, content and frequency), exercise (intensity, duration, type) and environment (enclosed versus free-living). Malnourishment of patients in ICUs is worryingly common, where one report notes that 43% of patients in one ICU were malnourished [113]. A study of 50 adults in intensive care demonstrated that a stunning 75% were under or over fed, when compared to indirect calorimetry [114]. The report notes that the severity of the underlying pathology greatly effects the energetic requirement of the patient. A modified version of the Harris–Benedict equation was used to calculate the total calorific need per day and adjusted daily according to the condition of the patient. Several shortcomings were noted, however, including prescription error and incorrectly judging the condition of a patient. The author notes that indirect calorimetry is considered the gold standard technique for determining energetic requirements; however, it is not widely available nor affordable. The need for a portable, simple and affordable indirect calorimeter is clear.

*Handheld calorimeters* are available on the market, but often are unable to meet the expectations set by the whole body calorimeter standard. The *MedGem* (Microlife Medical Home Solutions) is promoted as a handheld calorimeter for measurement of RMR. A comparison between the MedGem and traditional metabolic cart (Vmax29N) across 88 subjects was performed by Anderson [115]. The resting EE of 88 overweight or obese adults (BMI ≥ 25.0 and <40.0) was measured in random order. The MedGem was found to produce significantly higher measurements of EE than the indirect calorimetry. Overall, the mean EE across the volunteers was 1615.8 kcal/day, 1625.7 kcal/day and 1726.9 kcal/day measured by indirect calorimetry, the MedGem and calculated with predictive equations, respectively. On average, male subjects were overestimated by 150 kcal/day and female by 97.2 kcal/day using the MedGem method. It was concluded the MedGem provided a poor estimate of EE, and although traditional indirect calorimetry is preferred, the predictive equations were a valid assessment tool.

A further comparative study of the MedGem against an indirect calorimetry and predictive equations by Madden concluded that the handheld calorimeter provided estimates of resting EE that are less accurate than using predicative equations [116]. In this study of 36 healthy adults, it was found that the MedGem on average underestimated daily EE by 400 kcal/day. A notable problem with the MedGem is the adapted Weir formula, which is used to calculate EE. The device measures O_2_ and airflow, but not CO_2_. A constant value is set as the ratio between O_2_ consumed and CO_2_ produced (respiratory quotient (RQ) of 0.85). The RQ is of course intra-subject dependent; in the study by Anderson, measured RQ using indirect calorimetry found that the average coefficient for male subjects was 0.90 compared to 0.86 for females [115].

A mobile indirect calorimeter *Breezing* was reported as measuring the O_2_ consumed and CO_2_ produced, using a disposable single-use colorimetric cartridge [117]. The output from the metabolism calculations is displayed on a smartphone, using Bluetooth connectivity. The device was compared to reference sensors for O_2_ and CO_2_, with gas collected in a Douglas bag. The mobile device has only recently offered to consumers; thus, the device is not yet prominent in the literature, and external comparative studies against a range of subject groups are not available. In the study presented by Xian, 12 subjects (seven men, five women) aged between 21 and 33 years volunteered. The daily EE calculated by the new device was in the range of 1500–4000 kcal/day; all readings were within 10% of the Douglas bag method.

A research device for EE measurement was proposed by Vincent, to provide a portable alternative to respiratory chambers [118]. The device consists of affordable sensors, designed so the instrument can be distributed to patients, to allow recording of EE in a free-living environment. The device includes both an O_2_ sensor and a CO_2_ sensor, and it is proposed that the Weir Equation (1) is used to calculate daily EE from multiple short readings taken over a one-day period. A novel CO_2_ sensor was developed for use in the device [119]. The device enables a fast response sensor, but also a small size, ready for inclusion in a handheld analyser [120]. The device has shown promising results against synthetic gases in a laboratory environment, but at this stage in development, breath measurements have only been taken on a limited range of subjects.

## 5. Conclusions

A wide range of different diseases have been reported to be detected from exhaled human breath; suggesting that breath analysis is a promising tool for future healthcare practitioners. An increasingly common choice of transducer technology for detecting VOCs is acoustic wave devices (i.e., SAW, FBAR and quartz microbalances) with molecularly-sensitive thin coatings to create nano-sensor arrays. Quantifying accurately and reliably the gases on the exhaled breath of a patient is perhaps the next stage in this research field; however, the concept of *breathprinting* has been shown to offer a useful means of classifying subjects.

The measurement of our energy expenditure (EE) from breath is underrated as a tool for healthcare treatment. The rise in the proportion of the population becoming obese and the unprecedented cost to our healthcare services has sparked an interest in breath analysis. In this article, we have presented three different devices that enable hand-held metabolic rate monitoring through the exhaled levels of oxygen and carbon dioxide gas levels. Although no single device currently offers a revolutionary means of EE measurement, in the future, with the advent of smaller, low power consumption and faster sensors, this field is rapidly developing.

In conclusion, we believe that handheld and even wearable devices will be developed in the next few years, and they will bring the technologies of e-nose and calorimeters to monitor both our well-being and diagnose common diseases; perhaps the realization of Star Trek’s *tricorder*?

## Figures and Tables

**Table 1:** List of gases found in exhaled breath and their diagnostic purpose.

Gas	Inhaled	Normal Exhaled	Abnormal Exhaled	Healthcare Application/Diagnosis	Reference/s
Carbon dioxide	0.04%	3%–4%	-	Respiration monitoring, control of mechanical ventilator, capnography	[34,35,36]
Carbon Monoxide	0.25 ppm	0.5–2.1 ppm	16.7–29.3 ppm	Identification as smoker, chronic obstructive pulmonary disease (COPD)	[37,38,39]
			1.13 ppm	Severe sepsis	[40]
			5.6 ppm	Asthmatic patients	[41]
			4.4 ppm	Haemolysis	[42]
Nitric Oxide	<2 ppb	5.3–7.4 ppb	19.2–30 ppb	Asthma diagnosis	[43,44,45,46]
			16.3 ppb	Seasonal rhinitis	[47]
			12.9 ppb	Experimental influenza	[48]
			2.3–4.7 ppb	Cystic fibrosis	[49,50]
Hydrogen	0.5 ppm	<5 ppm	>16–20 ppm increase	Gastrointestinal diseases (small intestinal bacteria overgrowth, carbohydrate malabsorption), diarrhoea	[51,52,53,54,55]
Methane	1.7 ppm	<1 ppm increase	>16 ppm increase	Diverticulitis, constipation, irritable bowel syndrome	[56,57,58]

**Table 2:** Types of hydrogen breath tests and their diagnostic potential [52,54].

Test Substance	Indications from the Test
Fructose	Fructose malabsorption Irritable bowel syndrome Intolerance of sweets, honey or fruits Chronic inflammatory bowel diseases
Lactose	Estimation of oro-cecal transit time Small intestinal bacterial overgrowth Investigation of bloating Intolerance of milk, dairy products, pastries or chocolate Primary or secondary lactose intolerance
Glucose	Small intestinal bacterial overgrowth Exocrine pancreatic insufficiency Cirrhosis of the liver Secondary lactose intolerance Intolerance of sugar and sweets

**Table 3:** List of diseases and the VOCs found in relation to each.

Disease	VOCs	Reference
COPD	Isoprene, C 16 hydrocarbon, 4,7-dimethyl-undecane, 2,6-dimethyl-heptane, 4-methyl-octane, hexadecane	[67]
	Isoprene, acetic acid, benzaldehyde, benzene, nonadecane, toluene	[68]
	Benzaldehyde, limonene, 2-ethyl-1-hexanol, nonanal, menthone, menthol, decanal	[69]
	Decane, 6-ethyl-2-methyl-decane, benzene, butylated hydroxytoluene, limonene, 2-propanol	[70]
Lung Cancer	Acetone, acetonitrile, 2-3-dimethylbutane, hexane, limonene, pentane	[71]
	Ethyl benzene, hexane, trans-2-nonenal, pentane, 2-methyl pentane, heptanal	[72]
	Isobutane, methanol, ethanol, acetone, pentane, isoprene, benzene, toluene	[73]
	Toluene, o-xylene, styrene, 1-methyl-3-benzene, 2,3-dimethyl-hexane, 3-ethyl-3-methyl-2-pentanone	[74]
	Styrene, decane, isoprene, benzene, undecane, hexanol, heptanal	[75]
Breast Cancer	4-Methyl-2-heptanone, 2-nonanone, 2-dodecanone, 2,4-dimethyl-1-heptene, 2-xylene	[76]
	Cyclopropane, ethylidene, 1,4-pentadiene, 1,3-butadiene, 2-methyl	[77]
	3-methylhexane, decene, caryophyllene, naphthalene, trichloroethylene	[78]
	Heptanal, acetophenone, isopropyl myristate, 2-propanol	[79]
Chronic Liver Disease	Ammonia	[80]
	1-Decene, 1-octene, €-2-nonene	[81]
	Isoprene, carbon disulphide, dimethyl sulphide, pentane, ethane, acetone	[82]
Liver Cirrhosis	Methanol, 2-butanone, 2-pentanone, C8-kentone, sulfoxide-compound	[83]
	3-methylbutanal, propionic acid, octane, terpene, 3-carene, 1-hexadecanol	[84]
	Acetone, styrene, dimethyl sulphide, dimethylsilane, phenol, tetradecane	[85]
	Limonene, methanol, 2-pentanone	[86]
Colorectal Cancer	Ethanol, acetone, ethyl acetate, 4-methyl octane	[87]
	Nonanal, 4-methyl-2-pentanone, decanal, cyclohexane, 1,3-dimethylbenzene	[88]
	Cyclohexanone, 2,21-dimethyldecane, ethylaniline, cyclo-octylmethanol	[89]
Ovarian Cancer	Styrene, nonanal, 2-ethylhexanol, 3-heptanone, decanal, hexadecane	[90]
	Decanal, nonanal, styrene, 2-butanone, hexadecane	[91]

## Abbreviations

The following abbreviations are used in this manuscript:

MDPI	Multidisciplinary Digital Publishing Institute
CO_2_	Carbon dioxide
CO	Carbon monoxide
CF	Cystic fibrosis
DLW	Doubly-labelled water
EE	Energy expenditure
E-nose	Electronic nose
FBAR	Film bulk acoustic resonator
HR	Heart rate
ICU	Intensive care unit
MR	Metabolic rate
MS	Mass spectrometer
NO	Nitric oxide
O_2_	Oxygen
ppb	Parts per billion
ppm	Parts per million
SAW	Surface acoustic wave
SIT	Sprint interval training
VOC	Volatile organic compound
